# Non-aneurysmal non-traumatic subarachnoid hemorrhage: patient characteristics, clinical outcome and prognostic factors based on a single-center experience in 125 patients

**DOI:** 10.1186/1471-2377-14-140

**Published:** 2014-07-01

**Authors:** Juergen Konczalla, Johannes Platz, Patrick Schuss, Hartmut Vatter, Volker Seifert, Erdem Güresir

**Affiliations:** 1 Department of Neurosurgery, Johann Wolfgang Goethe-University Frankfurt am Main, Schleusenweg 2-16, 60528 Frankfurt am Main, Germany

**Keywords:** Non-aneurysmal, Aneurysm, Perimesencephalic, Non-perimesencephalic, Prepontine, Subarachnoid hemorrhage, SAH

## Abstract

**Background:**

Subarachnoid hemorrhage (SAH) is mainly caused by ruptured cerebral aneurysms but in up to 15% of patients with SAH no bleeding source could be identified. Our objective was to analyze patient characteristics, clinical outcome and prognostic factors in patients suffering from non-aneurysmal SAH.

**Methods:**

From 1999 to 2009, data of 125 patients with non-aneurysmal SAH were prospectively entered into a database. All patients underwent repetitive cerebral angiography. Outcome was assessed according to the modified Rankin Scale (mRS) (mRS 0–2 favorable vs. 3–6 unfavorable). Also, patients were divided in two groups according to the distribution of blood in the CT scan (perimesencephalic and non-perimesencephalic SAH).

**Results:**

106 of the 125 patients were in good WFNS grade (I-III) at admission (85%). Overall, favorable outcome was achieved in 104 of 125 patients (83%). Favorable outcome was associated with younger age (*P* < 0.001), good admission status (*P* < 0.0001), and absence of hydrocephalus (*P* = 0.001).

73 of the 125 patients suffered from perimesencephalic SAH, most patients (90%) were in good grade at admission, and 64 achieved favorable outcome.

52 of the 125 patients suffered from non-perimesencephalic SAH and 40 were in good grade at admission. Also 40 patients achieved favorable outcome.

**Conclusions:**

Patients suffering from non-aneurysmal SAH have better prognosis compared to aneurysm related SAH and poor admission status was the only independent predictor of unfavorable outcome in the multivariate analysis. Patients with a non-perimesencephalic SAH have an increased risk of a worse neurological outcome. These patients should be monitored attentively.

## Background

Spontaneous subarachnoid hemorrhage (SAH) is usually caused by rupture of an intracranial aneurysm. In up to 15% of patients with spontaneous SAH, no bleeding source can be identified despite of repetitive radiological imaging [1-5]. In 20 to 70% of patients with angiography negative SAH the blood distribution is described as perimesencephalic or prepontine [6]. Patients with perimesencephalic SAH are considered to achieve a good outcome and to have a lower risk of rebleeding [6,7]. However, data on patients suffering from spontaneous non-aneurysmal SAH is limited. There are a few studies reporting on a limited number of patients [2,8-12]. Therefore, the aim of the present study was to investigate the clinical course and outcome in patients suffering from non-aneurysmal spontaneous SAH.

## Methods

After exclusion of patients with traumatic SAH we reviewed 1046 consecutive patients with SAH admitted in our department between 1999 and 2009. SAH was confirmed on computed tomography (CT) and/or lumbar puncture. We identified 125 patients (12%) with a spontaneous non-aneurysmal SAH after diagnostic workup. Information, including patient characteristics, treatment, radiological features, and outcome was prospectively entered into a computerized database.

In our hospital algorithm all patients with SAH underwent angiography and since 2002 at least four-vessel 3D digital subtraction angiography (DSA) to rule out intracranial sources for SAH. In case of a negative initial angiography, DSA was repeated after 14 days. Additionally, magnetic resonance imaging (MRI) of the spine was performed to rule out any spinal bleeding sources. In patients without typical perimesencephalic distribution of blood a third DSA was performed 3 months after SAH. Patients were stratified according to the distribution of blood into two groups, perimesencephalic (PM) and non-perimesencephalic (NPM) SAH.

The study was approved by the local ethics committee of Goethe-University hospital Frankfurt.

**Figure 1:**
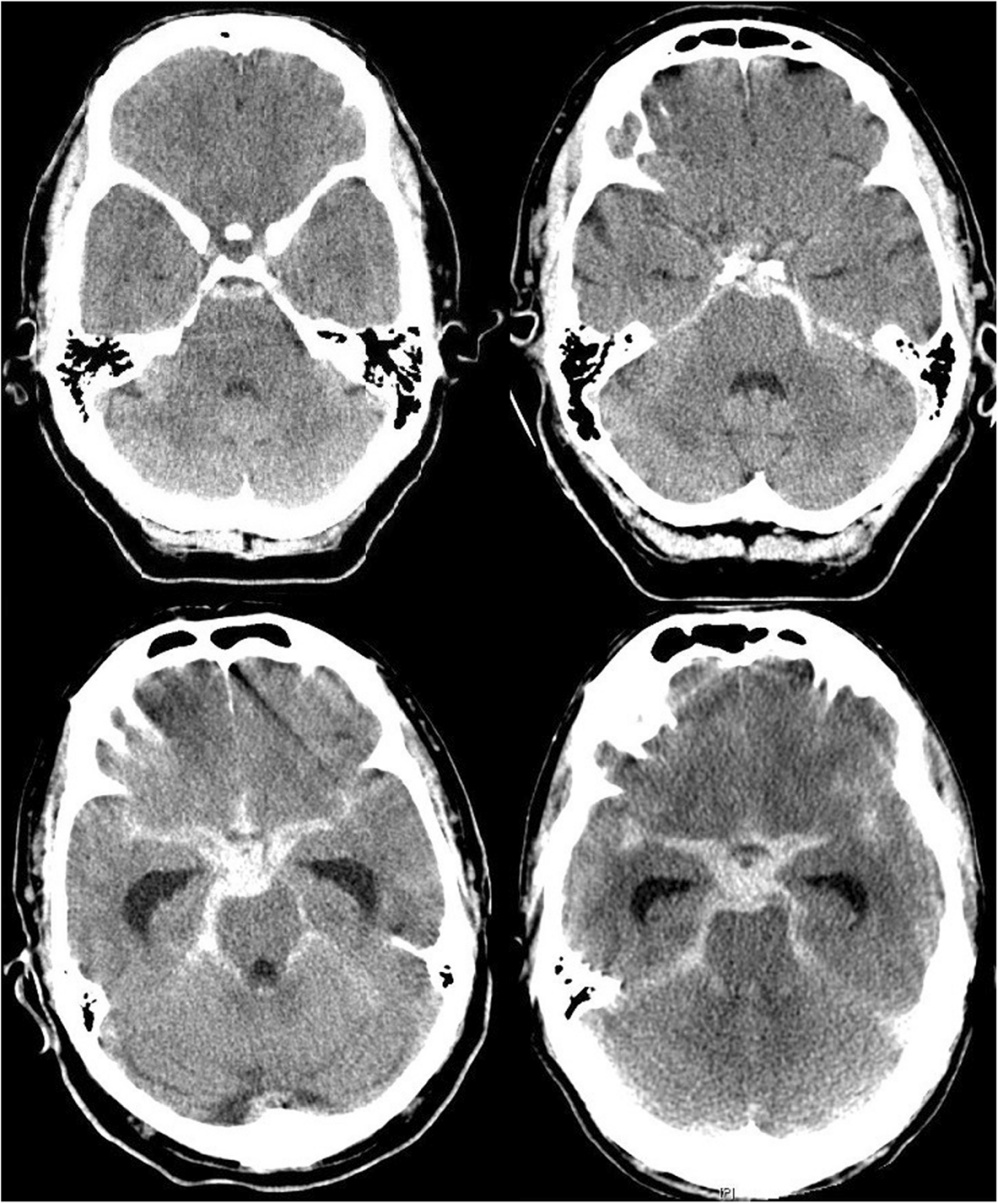
**Perimesencephalic and non-perimesencephalic SAH.** In the upper row CT scans of two patients with perimesencephalic SAH are illustrated. Whereas in the top left-hand the center of the hemorrhage is located anterior the brainstem, in the top right-hand corner the hemorrhage is located more left-sided. In the lower row CT scans of two patients with non-perimesencephalic SAH are illustrated. In both patients the bleedings are not only located anterior the midbrain, but also enlarging into the sylvian and interhemispheric cisterns. Also in contrast to the upper row these patients had a hydrocephalus with enlargement of the temporal horns.

We defined a perimesencephalic hemorrhage according to van Gijn et al. [7] and Rinkel et al. [13]. Hemorrhages located in front of the brain stem, mainly in the interpeduncular cistern, with or without extension to the ambient, chiasmatic and horizontal part of the Sylvian cisterns, were classified as perimesencephalic SAH, whereas in non-perimesencephalic hemorrhages blood was not located mainly in the interpeduncular cistern, but in the Sylvian cistern, interhemispheric cistern, convexity or a CT-negative and lumbar puncture positive bleeding (see Figure 1). Our treatment protocol includes the application of nimodipine in all patients with SAH from the day of admission and has been described in detail previously [14]. Patients were monitored daily with transcanial Doppler ultrasound (TCD) at least up to day twelve after bleeding. Patients with a non-perimesencephalic SAH without suspicion of delayed cerebral ischemia (DCI) and intubated patients with a perimesencephalic SAH routinely received on day 7 ± 2 after ictus a CT or MRI. We defined DCI based on the Vergouwen-definitions [15]; in brief, the occurrence of focal neurological impairment, which cannot be attributed to other causes. For comatose and sedated patients, in which a DCI couldn’t be monitored, instead of DCI a proximal arterial diameter reduction of more than 66% was defined as cerebral vasospasm. In our hospital algorithm patients received an invasive diagnostic and therapeutic management (angioplasty and/or intraarterial nimodipine) [14], but in this cohort only one patient received an angioplasty with two intraarterial nimodipine therapies, and another patient received an one-time intraarterial nimodipine treatment.

Patients were divided into good grade (WFNS grades I – III) versus (vs.) poor grade (WFNS IV – V) on admission. Outcome was assessed according to the modified Rankin Scale (mRS) and stratified into favorable (mRS 0–2) vs. unfavorable (mRS 3–6) after 6 months.

In non-comatose patients hydrocephalus was defined as clinical deterioration (like deterioration from somnolence to stupor) and enlargement of the temporal horns of more than 2 mm. In comatose patients the enlargement of the temporal horns was defined as hydrocephalus and normally an external ventricular drainage was placed.

### Statistical analysis

Unpaired t-test was used for parametric statistics. Categorical variables were analyzed in contingency tables using the Chi^2^ test. Results with a *P* value < 0.05 were considered statistically significant. Variables with significant probability values on univariate analysis were considered as potentially independent variables on multivariate analysis. A backward stepwise method was used to construct multivariate logistic regression models with the inclusion criterion of *P* < 0.05. All calculations were made with standard commercial software (SPSS Institute, Inc.).

## Results

### Patient characteristics

**Figure 2:**
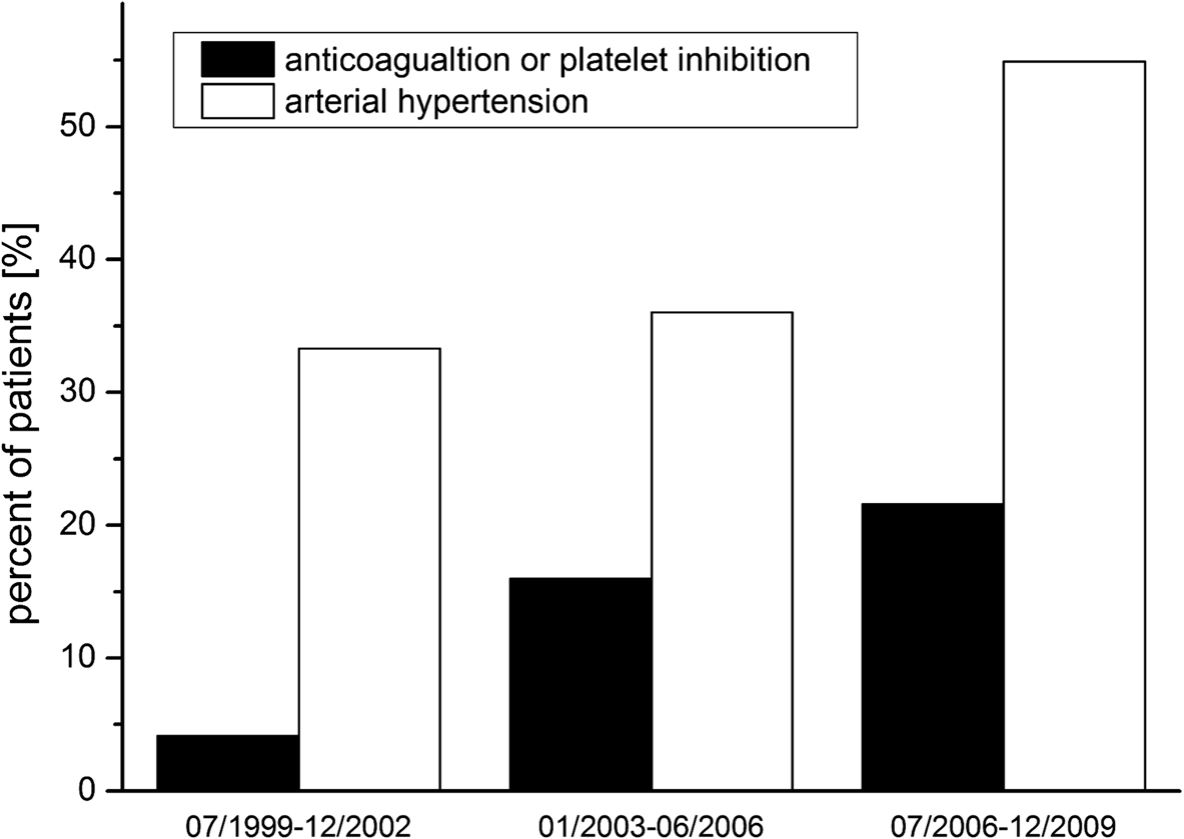
**Rate of arterial hypertension and anticoagulation.** From 07/1999 to 12/2009 we had an increasing rate of patients with arterial hypertension (from 33% to 55%). Also the rate of patients with systemic anticoagulation and/or platelet inhibition increased in this period from 4% to 22%.

After diagnostic workup in 125 of 1046 patients (12%) with spontaneous SAH no bleeding source was identified and these patients were included in this study. The mean age was 56 years. Men were more frequently affected by non-aneurysmal SAH (70%). Patient characteristics are detailed in Table 1. From 07/1999 to 12/2002 24 patients had a non-aneurysmal SAH. From 1/2003 to 6/2006 50 patients (108% increase in comparison to the period before) had a non-aneurysmal SAH and from 07/2006 to 12/2009 51 patients (113% increase) had a non-aneurysmal SAH. Also the rate of patients with systemic anticoagulation or platelet inhibition increased from 4% to 22% over the periods (see Figure 2).

**Table 1:** Patient characteristics

**Characteristics**	**Non-aneurysmal SAH (% of total pat.)**	**Perimesencephalic SAH (%)**	**Non-perimesencephalic SAH (%)**	*P*-value*
No. of patients	125	73 (58%)	52 (42%)	NS
Mean age +/- SD	56.4 ± 13.1	54.6 ± 12.1	58.8 ± 14.2	NS
Female sex	38 (30%)	18 (25%)	20 (38%)	NS
Mean WFNS at admission	II	II	II	
WFNS I-III at admission	106 (85%)	66 (90%)	40 (77%)	NS
Early hydrocephalus	36 (29%)	18 (25%)	18 (35%)	NS
Arterial hypertension	54 (43%)	28 (38%)	26 (50%)	NS
Mean mRS	1	1	2	
Favorable outcome	104 (83%)	64 (88%)	40 (77%)	NS
Death after 6 months	12 (10%)	5 (7%)	7 (13%)	NS
VP-shunt	12 (10%)	5 (7%)	7 (13%)	NS

*no.* number; *pat.* patients; *WFNS* World Federation of Neurological Societies; *mRS* modified Rankin scale; *VP* ventriculoperitoneal.

*unpaired t-test for parametric statistics and Chi^2^ test for categorical variables; *NS* not significant (p > 0.05).

### Clinical presentation

Overall, 106 patients (85%) were in good clinical condition at admission.

40 of 52 patients (77%) suffering from non-perimesencephalic SAH had good admission status compared to 66 of 73 patients (90%) suffering from perimesencephalic SAH (*P* = 0.07). In 54 patients (43%) an arterial hypertension (≥140 mmHg systolic blood pressure) was diagnosed (see Table 1). Like for anticoagulants the rate of patients with hypertension increased over the periods (from 33% to 55%) (see Figure 2).

### Imaging results

Overall, initially in 137 patients a non-aneurysmal SAH was detected by CT or lumbar puncture. In twelve of these 137 patients (9%) a bleeding source was detected by DSA (11 patients) or MRI (1 patient). Five aneurysms of the anterior circulation, four aneurysms of the posterior circulation, one cerebellar AVM and one AV-fistula were detected in the following DSA and a spinal pathology in the MRI. These patients were excluded from this study. Overall, 52 of the 125 patients (42%) suffered from non-perimesencephalic, and 73 patients (58%) from perimesencephalic SAH (see Figure 1 and Table 1). 12 patients (10%) had a CT-negative/lumbar puncture positive SAH. The initial angiograms of these patients were all negative for a vasoconstriction. 38% of the patients with a non-perimesencephalic SAH had a SAH according Fisher type 3 [16]. Only 2 patients (4%) had a convexity-located SAH. Respectively, one woman with a high fronto-parietal SAH and one man with a temporo-mesial SAH.

### Early vs. late hydrocephalus

Overall, 36 patients (29%) suffered from early hydrocephalus at admission and underwent a placement of a ventricular drain. 12 of the 36 patients (10%) developed posthemorrhagic hydrocephalus and underwent a placement of a ventriculoperitoneal shunt (VP-shunt). 7 patients (13%) with non-perimesencephalic SAH vs. 5 patients (7%) with perimesencephalic SAH developed late hydrocephalus over the course of treatment.

23 patients (22%) with favorable outcome vs. 13 patients (62%) with unfavorable outcome suffered from early hydrocephalus (*P* < 0.001, OR = 0.2, 95% CI = 0.1–0.5; Table 2).

**Table 2:** Outcome of non-aneurysmal SAH

**Characteristics**	**Favorable outcome**	**Unfavorable outcome**	**OR (95% CI)**	*P*-value*
Number of patients	104 (83%)	21 (17%)		
Mean age +/- SD	54.5 ± 12.7	65.7 ± 11.4	11.2 (5–17)	< 0.001
Good admission status	96 (92%)	10 (48%)	13.2 (4–40)	< 0.0001
Early hydrocephalus	23 (22%)	13 (62%)	0.17 (0.06-0.47)	< 0.001
			5.7 (2.1-15)	
Arterial hypertension	45 (43%)	9 (43%)		NS
Female sex	29 (28%)	9 (43%)		NS

*unpaired t-test for parametric statistics and Chi^2^ test for categorical variables; *WFNS* World Federation of Neurological Societies, *OR* odds ratio; *CI* confidence interval; *NS* not significant (p > 0.05).

### Neurological outcome and delayed cerebral ischemia

Overall, favorable outcome was achieved in 104 patients (83%).

More precise, this favorable outcome was achieved in 64 patients (88%) of PM-SAH vs. 40 patients (77%) in NPM-SAH (*P* = 0.18). 5 patients in PM-SAH (7%) vs. 7 patients in NPM-SAH (13%) died during the observation period (*P* = 0.35) (Table 1). In 18 patients (14%) a delayed cerebral ischemia (DCI) [15] was observed. Patients with a NPM-SAH suffered more often a DCI (19%) compared to patients with PM-SAH (11%), but no statistical significant difference between these groups could be determined (*P* > 0.05, OR = 0.5, 95% CI = 0.2-1.4). Also sex and arterial hypertension did not influence the outcome.

Patients, who achieved favorable outcome were younger compared to patients, who achieved unfavorable outcome (55 vs. 66 years; *P* < 0.001).

Patients presented in a good clinical status at admission achieved significantly more often a favorable outcome compared to patients with a poor admission status (92% vs. 48%; *P* < 0.0001, OR = 13.2, 95% CI = 4–40; Table 2).

### Multivariate analysis

Of those variables that influenced clinical outcome of patients at 6 months after discharge in the univariate analyses (age > 65 years, hydrocephalus and poor admission status), only the variable “poor admission status” (*P* < 0.001, OR = 13.2, 95% CI = 4–40) remained significant as an independent predictor of unfavorable outcome in the multivariate regression model (Nagelkerke`s R^2^ = 0.26).

## Discussion

In up to 15% of patients suffering from SAH, no source of bleeding could be identified [2,6,8,17-19]. The majority of patients with non-aneurysmal SAH are in good clinical condition at admission [2,6,8,18,19]. Non-aneurysmal and especially perimesencephalic SAH is often associated with favorable outcome compared to aneurysmal SAH [1,7,13,20]. However, data on patients with spontaneous non-aneurysmal SAH are commonly based on case series and is therefore scarce [2,6,8-10,17,19,21].

We therefore analyzed our neurovascular database concerning patients suffering from non-aneurysmal SAH. In the present series, 12% of patients (n = 125) with SAH had no source of bleeding after repeated cerebral angiograms and MRI of the spine.

### Clinical presentation

Patients suffering from non-aneurysmal SAH are usually in good clinical condition [2,6,8,18,19]. This could be confirmed in the present study, in which 85% of the patients were in a good status at admission (WFNS grade I - III). However, patients with a perimesencephalic (PM) SAH were in a better status at admission compared to patients with a non-perimesencephalic (NPM) SAH (90% vs. 77%), not reaching statistically significance (*P* < 0.07). In both subgroups, PM-SAH and NPM-SAH, men are affected more likely compared to women (for PM-SAH in 75% and for NPM-SAH in 62%).

Despite the technological improvements of DSA, we found increasing cases of angiography-negative SAH in our analysis. This could be explained by the increasing numbers of patients with systemic anticoagulation or platelet inhibition (see Figure 2). However, data for antithrombotic therapy in non-aneurysmal SAH is scarce, but describing higher blood amount and rebleeding rates [22-24]. A further reason for the growing numbers of angiography-negative SAH could be the increasing numbers of arterial hypertension (see Table 1).

### Imaging results

An intracranial source of hemorrhage was identified in eleven of 137 patients (8%) by DSA. In one patient (1%) after aneurysm-negative DSA in the spinal MRI a pathology as source of hemorrhage was identified. Compared to other published data with series of more than 50 patients this rate is very similar (7%-18%) [5,17,25,26]. Some studies described lower rates, but repeated angiography were done in series with less than 50 patients [21,27].

A recently published study reported a significantly lower rate of rebleeding in patients with perimesencephalic SAH compared to patients with NPM-SAH [28]. And rebleeding is the most important preventable cause of unfavourable outcome in patients suffering from spontaneous SAH [29]. However, in patients with aneurysm-negative baseline-DSA, repeat angiography is necessary in order to detect a potential bleeding source and to prevent rebleeding [25,30]. Due to the small, but substantial risk of neurological worsening after DSA, in PM-SAH a non-invasive angiography by MRI or CT might be sufficient [3-5,31]. In patients with a Fisher type 3 pattern of hemorrhage a false-negative DSA rate up to 46% was found [26], therefore a repeated DSA should be performed in NPM-SAH [7,26,31]. In our treatment protocol all patients receive two DSA and MRI of the whole spine, resulting in only one rebleeding after this diagnostic work-up. In twelve patients (9%) a bleeding source was detected. For sulcal convexity SAH in up to 87% of the patients the combination of MRI and DSA could identify the aetiology of bleeding, like reversible cerebral vasoconstriction syndrome (RCVS or Call-Fleming), cerebral amyloid angiopathy or posterior reversible encephalopathy syndrome (PRES), which represented more than 50% of etiological mechanisms [12]. Unfortunately, in our two cases with sulcal convexity SAH an aetiology wasn’t diagnosed. In the present series after diagnostic work-up 125 patients had a spontaneous angiogram-negative SAH.

### Early vs. late hydrocephalus

In both groups approximately 30% of the patients had hydrocephalus at admission, which is higher compared to other series (5 - 25%) [6,8,32]. Despite the higher rate of early hydrocephalus, the necessity of permanent shunt placement (10%) was similar to previous reports (3 – 13.5%) [6,8,21,32].

However, incidence of shunt dependency did not differ significantly in patients with PM-SAH compared to patients with NPM-SAH.

### Neurological outcome

Non-aneurysmal and especially perimesencephalic SAH is associated with favorable outcome compared to aneurysmal SAH [1,7,13,20]. In the present series, favorable outcome was also achieved in a high number of patients (83%), with a trend towards a better outcome in the PM-SAH group (88% vs. 77% in NPM-SAH) and also a trend towards a lower incidence of DCI (11% in PM-SAH vs. 19% in NPM-SAH). Nayak et al. and Woznica et al. also identified different outcomes of non-aneurysmal SAH according to the blood distribution [18,33]. Although it is taught, that there are only exceptions to a cerebral vasospasm (CVS), in this study 11% of the patients with PM-SAH developed a DCI. Severe vasospasms also occurred in non-aneurysmal SAH, requiring endovascular intervention [34]. Therefore, clinical outcome of patients with a PM-SAH seems to be better. This also may be due to the lower risk of vasospasm or cerebral ischemia. In the present univariate analysis, early hydrocephalus and poor admission status were significantly associated with unfavorable outcome. Dalyai et al. report a higher risk for hydrocephalus and cerebral vasospasm in the NPM-SAH group compared to PM-SAH patients [25]. However, in the multivariate analysis the only independent predictor for unfavorable outcome in patients with non-aneurysmal SAH was “poor admission status” (*P <* 0.0001, OR = 13.2, 95% CI = 4–40).

The sex-specific prevalence for a non-aneurysmal SAH is inconsistent and varies from female predominance [6,19], no predominance [32], to male predominance [2,8,21], like in our series, but this doesn’t influence the outcome (see Table 2).

### Comparison with aneurysmal SAH

In the present series of non-aneurysmal SAH the mean age (56 years) was similar compared to data of the International Subarachnoid Aneurysm Trial (ISAT) [35] (52 years) or the data of the Barrow Ruptured Aneurysm Trial (BRAT) [36] (53–54 years).

Unlike aneurysmal SAH in the present series more men than women were affected by non-aneurysmal SAH; detailed in Table 3.

**Table 3:** Comparison of non-aneurysmal SAH vs. ISAT/BRAT-data

**Characteristics**	**ISAT 2002**	**OR (95% CI)**	*P*-value *	**non-aneurysmal SAH**	**BRAT 6mon**	**OR (95% CI)**	*P*-value *
No. of patients	2143			125	471		
Mean age	52			56.4 ± 13.14	53 to 54		
Male gender	799 (37%)	3.9 (2.6 - 5.7)	< 0.0001	87 (70%)	139 (30%)	5.5 (3.6 - 8.4)	< 0.0001
WFNS/HH I-III at admission	2018 (94%)	0.35 (0.2 - 0.6)	< 0.0001	106 (85%)	380 (81%)		NS
				***Outcome***			
	**ISAT 2002**				**BRAT 6mon**		
**No. of patients**	1594			125	341		
Favourable outcome (mRS 0–2)	1161 (73%)	1.85 (1.1-3.0)	0.011	104 (83%)	239 (70%)	2.1 (1.3-3.6)	< 0.01
Death	145 (9%)	1.1 (0.6-2.0)	NS	12 (10%)			
	**ISAT 2009**						
**No. of patients**	1644			125			
DID /DCI	415 (25%)	2.0 (1.2 - 3.3)	< 0.01	18 (14%)			

*unpaired t-test for parametric statistics and Chi^2^ test for categorical variables vs. ISAT/BRAT; *WFNS* World Federation of Neurological Societies, *OR* odds ratio; *CI* confidence interval; NS: not significant (p > 0.05); ISAT 2002: comparison with data from Molyneux et al. [35]; ISAT 2009: comparison with data from Rivero-Arias et al. [37]; BRAT 6mon: comparison with 6 months outcome data from Spetzler et al. [36].

*DID* delayed ischemic neurological deficit according to the ISAT 2009 data [37]; *DCI* delayed cerebral ischemia, according the Vergouwen definitions [15].

Although we didn’t know the cause and subsequent pathophysiology of a non-aneurysmal SAH, the patients had a better outcome compared to aneurysmal SAH (*P* ~ 0.01; Table 3). We also detected a reduced risk of DCI compared to DID in ISAT (*P* < 0.01; Table 3), which could be one reason for the better outcome. In the light of expected higher number of DCI (including angiographic severe vasospasm) than DID (only neurological deficit and separate data for severe angiographic vasospasm) this fact may be reinforced, when angiographic vasospasm will be excluded from the present data. Especially patients with a NPM-SAH, and a trend towards higher risk of DCI compared to PM-SAH, should be monitored attentively.

### Limitations

The study has several limitations. Statistical analysis was retrospectively performed and conducted from a single center, but the data was collected prospectively. Due to the retrospective design there are the typical restrictions such as the lack of data not documented initially in the medical records. Most of our patients are referred to us from other hospitals, therefore the documentation of the initial blood pressure is often missing and only arterial hypertension (≥140 mmHg systolic blood pressure) could be examined. Also a control group is absent. The follow-up examination was conducted 6 months after ictus. To reduce this limitation a comparison with two prospective multicenter studies was performed, also using follow-up at least 6 months after ictus [35-37]. Despite the good outcome, defined by mRS 0–2, a further investigation with long-term outcome using a more complex scale, dividing functional, social and psychological outcome, would be of great interest.

## Conclusion

Perimesencephalic and non-perimesencephalic SAH have similar presentation status. Favorable outcome is achieved in most patients. Poor admission status was the only independent predictor of unfavorable outcome in patients with spontaneous non-aneurysmal SAH in the multivariate analysis. In this large study (for non-aneurysmal SAH) a significantly different clinical outcome was identified compared to aneurysmal SAH. Patients with non-aneurysmal SAH have significantly less DCI compared to aneurysmal SAH, but patients with PM and NPM-SAH also developed DCI. An early hydrocephalus seems not to worsen the outcome, if sufficient and early treated. Patients with a NPM-SAH appear to have an increased risk for DCI and for a worse neurological outcome compared to PM-SAH. However, only a trend towards significance could be calculated. Therefore, patients with a NPM-SAH should be monitored attentively.

## Competing interests

The authors declare that they have no competing interests. The authors have no personal financial or institutional interest in any of the drugs, materials, or devices described in this article.

## Authors’ contributions

JK, JP, PS acquired and analyzed the data. JK performed the statistical analysis and drafted the manuscript. JK, EG, HV, VS interpreted the data and contributed to critical revision of the manuscript for important intellectual content. EG performed critical supervision of the manuscript. All authors read and approved the final manuscript.

## Pre-publication history

The pre-publication history for this paper can be accessed here:

http://www.biomedcentral.com/1471-2377/14/140/prepub
